# Verification of the Three-Step Model in Assessing the Pathogenicity of Mismatch Repair Gene Variants

**DOI:** 10.1002/humu.21409

**Published:** 2010-11-30

**Authors:** Minttu Kansikas, Reetta Kariola, Minna Nyström

**Affiliations:** Department of Biosciences, Genetics, University of HelsinkiHelsinki, Finland

**Keywords:** Colorectal cancer, CRC, HNPCC, Lynch syndrome, VUS, *MLH1*, *MSH2*, *MSH6*

## Abstract

In order to assess whether variations affecting DNA mismatch repair (MMR) genes are pathogenic and hence predisposing to Lynch syndrome (LS), a three-step assessment model has been proposed. Where LS is suspected based on family history, STEP1 is dedicated to the identification of the causative MMR gene and the variation within it. Thereafter, in STEP2 of the assessment model, the effect of the variation on the function of the protein is assessed in an in vitro MMR and in silico assays. Where LS cannot be confirmed or ruled out in STEP2, the more specific biochemical laboratory assays such as analyzing the effect of the variation on expression, localization, and interaction of the protein are required in STEP3. Here, we verified the proposed three-step assessment model and its ability to distinguish pathogenic MMR variations from variants of uncertain significance (VUS) by utilizing the clinical as well as the laboratory and in silico data of 37 *MLH1*, 26 *MSH2,* and 11 *MSH6* variations. The proposed model was shown to be appropriate and proceed logically in assessing the pathogenicity of MMR variations. In fact, for MMR deficient *MSH2* and *MLH1* variations the first two steps seem to be sufficient as STEP3 provides no imperative information concerning the variant pathogenicity. However, the importance of STEP3 is seen in the assessment of MMR proficient variations showing discrepant in silico results as their pathogenicity cannot be confirmed or ruled out after STEP2. *MSH6* variations may be applicable to the model if appropriate selection in terms of ruling out *MLH1* and *MSH2* variations and *MLH1* promoter hypermethylation is ensured prior to the completion of STEP2. In conclusion, taking into consideration the susceptibility gene the three-step model can be utilized in an appropriate and efficient manner to determine the pathogenicity of MMR gene variations. Hum Mutat 32:107–115, 2011. © 2010 Wiley-Liss, Inc.

## Introduction

Lynch syndrome (LS, often referred to as hereditary nonpolyposis colorectal cancer syndrome; HNPCC; MIM♯ 120435) is highly associated with autosomal dominant inheritance of mutations in genes fundamental to the DNA mismatch repair (MMR) mechanism. The most frequently affected genes include *MLH1* (MIM♯ 120436, RefSeq NM_000249.3), *MSH2* (MIM♯ 609309, RefSeq NM_000251.1), *MSH6* (MIM♯ 600678, RefSeq NM_000179.2), and *PMS2* (MIM♯ 600259, RefSeq NM_000535) whose germline variations are reported in the LOVD database (http://www.insight-group.org/; http://www.lovd.nl/). Although, the majority of mutations affecting MMR genes are truncating, a significant proportion of mutations result in a single amino acid substitution or an in-frame deletion and are difficult to distinguish from harmless polymorphisms. Such alterations are often referred to as variants of uncertain significance (VUS) [Goldgar et al., 2008] due to the uncharacterized effect of the variation on the function of the polypeptide.

LS-associated tumors generally occur in the colon; nevertheless, a variety of extracolonic carcinomas, especially those of the endometrium, are also frequently observed. The mean age of cancer onset in LS is significantly lower than that of sporadic colorectal cancer (CRC) [Lynch and de la Chapelle, 1999] based on the fact that in LS, an individual has already inherited susceptibility through a mutated allele and only needs a second hit in a somatic cell to lose MMR activity and start tumorigenesis. Hence, LS tumors are characterized by the lack or lowered level of a causative MMR protein as well as impaired DNA repair causing microsatellite instability (MSI) [Aaltonen et al., 1993]. The wide variety of clinical phenotypes complicates LS diagnostics and several clinical guidelines have been established to distinguish LS families from the general CRC burden. Currently, the clinical diagnosis of LS greatly relies on the Amsterdam criteria (AC) [Vasen et al., 1991, 1999] or the revised Bethesda guidelines [Umar et al., 2004], which take into account the age of cancer onset, the number and segregation of affected individuals in a family, and the level of MSI. However, many putative LS families do not fit these criteria and could be confirmed as LS families only by characterizing a pathogenic germline MMR gene mutation in them.

The first clinical step in diagnosing LS associated tumors includes immunohistochemistry (IHC) and MSI analysis followed by mutation analysis dictated by the IHC and MSI results. Hampel et al. [2005] have proposed a strategy for screening LS by analyzing all four MMR genes (*MLH1*, *MSH2*, *MSH6,* and *PMS2*) together with the potential hypermethylation of the *MLH1* promoter region. When a variation with a known defect is found, LS can be confirmed or in the absence of a MMR gene variation, ruled out.

Based on a similar approach (STEP1), Couch et al. [2008] have proposed a decision tree for the in vitro analysis of MMR VUS found in putative LS families. This model utilizes data from incompletely validated assays supplemented with data derived from other sources for classification of VUS for clinical purposes. More specifically, data derived from an in vitro MMR and in silico analyses should be considered upon the identification of a VUS (STEP2). Variations showing MMR deficiency in these assays indicate LS, whereas variations with no apparent MMR deficiencies require a selection of biochemical assays for further characterization of the effect of the variation on the protein expression or function (STEP3).

Here, we aim to verify the ability of the proposed three-step model in assessing pathogenicity with the data of 74 MMR gene VUS including results of tumor pathologic, genetic, biochemical, and in silico analyses.

## Materials and Methods

### *MLH1*, *MSH2,* and *MSH6* Variations and Clinical Data

This study comprises 37 *MLH1* (NM_000249.3) [Christensen et al., 2009; Raevaara et al., 2005], 26 *MSH2* (NM_000251.1) [Christensen et al., 2009; Kariola et al., 2003; Ollila et al., 2006, 2008], and 11 *MSH6* (NM_000179.2) [Kariola et al., 2002, 2003, 2004] variations. Nucleotide numbering reflects cDNA with + 1 corresponding to the A of the ATG translation initiation codon in the reference sequence, according to journal guidelines (http://www.hgvs.org/mutnomen). The family history and the data of mutation, MSI and IHC analysis were mainly collected through an international LS collaboration. Because the 74 VUS included in the study were found by many research groups, different methods were used for mutation detection. The alterations are distributed over most of the known functional domains of the respective proteins as seen in the schematic representations presented in the results section. *MLH1* variations tend to cluster either at the amino terminal or carboxyl terminal of the protein, whereas *MSH2* and *MSH6* variations are dispersed throughout the length of the proteins, with preferential location in functional domain clusters seen particularly in the connector and ATPase domains of MSH2. The variations were constructed by site-directed mutagenesis and coexpressed with their native heterodimerization partners in *Spodoptera frugiperda* (*Sf9*) insect cells for protein production [Nyström-Lahti et al., 2002].

The VUS chosen for this study are from putative LS families either fulfilling the AC, or from families not fulfilling AC but presenting LS tumors at an abnormally low age, or with an excessive amount of LS cancers occurring within the family. In fact, of the 37 *MLH1* variations, at least 27 are associated with a mean age of onset below 50 years even though the ACI are only fulfilled by families associated with 17 of the variations. Nineteen out of 26 *MSH2* variations are associated with a mean age of onset under 50 years and approximately half of all the *MSH2* variations were found in families fulfilling ACII. *MSH6* variations chosen for functional assessment were mainly picked from the LOVD database. The families with *MSH6* variations do not fulfill AC, but instead, all but two variations have been linked to the MSI-high (MSI-H) tumor phenotype. The mean age of tumor onset of *MSH6* VUS carriers is 59.

### Functional Analyses

The 74 MMR gene VUS included in the verification study were functionally characterized in our previous studies [Christensen et al., 2009; Kariola et al., 2002, 2003, 2004; Ollila et al., 2008; Raevaara et al., 2005]. Studies were typically mandated by clinical questions and ambiguities tracking the variation, tumor pathology data, or in some cases database information. The present study is based on assessments performed in those studies, where functionality of each variation was evaluated in comparison to the performance of the corresponding wild-type (wt) protein and functionally deficient negative controls. Assay results were composed of a minimum of three independent experiments and a repeatedly seen decrease in functionality compared to the corresponding result of WT protein indicated pathogenicity.

The applied in vitro MMR assay [Nyström-Lahti et al., 2002] uses a homologous human MMR system to study the ability of the variant protein to repair a G·T mispair. The standard deviation of the repair efficiency of the deficient variant protein remained below that of the WT protein. To assess the effect of the variation on protein expression, MSH2 and MSH6 variant proteins were expressed in *Sf9* insect cells [Kariola et al., 2002], where as MLH1 proteins were expressed in 293T human cells. Thereafter, the expression levels of the VUS proteins were compared with that of WT protein by Western blot analysis [Raevaara et al., 2003]. Results from interaction studies are based on coimmunoprecipitation and subsequent Western blot analysis of the variant protein with its native heterodimerization partner [Kariola et al., 2002; Raevaara et al., 2003]. To study whether the *MLH1* variations affect the subcellular localization, VUS cDNAs were fused with the fluorescent protein EGFP cDNA and transiently expressed in 293T cells [Raevaara et al., 2005]. The variants acting like WT MLH1 were classified as normal in the localization study.

### In Silico Analysis by SIFT and MAPP-MMR

In silico methods take a computational approach to identify highly conserved areas of a gene through a multiple sequence alignment analysis across numerous species, and thereafter, deduce possible functional defects of a variation. Several prediction algorithms are available for in silico analyses. Here, the sorting intolerant from tolerant (SIFT) [Ng and Henikoff, 2001] (http://sift.jcvi.org/) and the multivariate analysis of protein polymorphism (MAPP-MMR) [Chao et al., 2008; Stone and Sidow, 2005] (http://mendel.standofrd.edu/SidowLab/) programs were chosen for in silico assessment of the 74 VUS due to their high sensitivity and specificity [Tavtigian et al., 2008b]. The results from previously done SIFT analyses for *MLH1* and *MSH2* VUS [Christensen et al., 2009; Ollila et al., 2006, 2008; Raevaara et al., 2005] were complemented with results of *MSH6* VUS obtained from the LOVD database (http://www.lovd.nl/). The MAPP-MMR analysis was performed here for the *MLH1* and *MSH2* variations but was not available for *MSH6* variations. Neither of the programs can be applied to in frame deletions.

### Verification of the Three-Step Model

**Figure 1:**
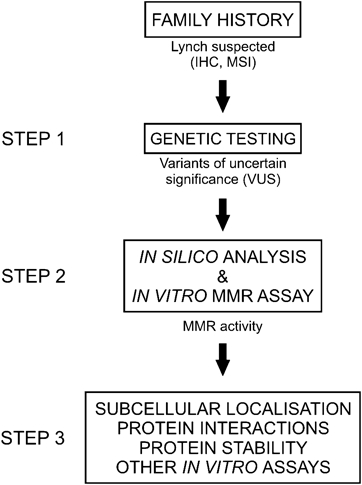
A three-step decision tree proposed to facilitate the functional assessment of VUS. (Modified from Couch et al. [2008].)

To assess the pathogenicity of VUS, a three-step model proposed by Couch et al. [2008] was applied (Fig. 1). The model acknowledges the importance of appropriate VUS identification by emphasizing the use of family history, MSI, and IHC data to ultimately identify the VUS by genetic testing analysis in STEP1. Upon the identification of a VUS, STEP2 consists of in vitro MMR and in silico analyses. MMR deficiency demonstrated by STEP2 confirms LS, whereas in the case of MMR proficient variations, a panel of biochemical assays is recommended in STEP3.

The 74 MMR VUS included in this study are found in families suspected to have LS, and hence compose an appropriate and realistic panel of variations for the verification of the three-step assessment model. Each variation is taken through the model and the assays constituting each of the steps. Results indicating pathogenicity are distinguished from ones indicating no effect of the variation. Discrepancies between individual tumor data (IHC and MSI) as well as between results from different in silico programs are marked.

STEP1 of the model proposed by Couch et al. [2008] is represented by the family history and tumor pathology data of the proband leading to mutation analysis. Tumors with two or more unstable Bethesda panel markers [Umar et al., 2004] were considered to have a high degree of MSI, and the lack or reduction of the MMR protein in IHC was considered to indicate protein deficiency.

Upon the identification of a VUS, STEP2 of the assessment model [Couch et al., 2008] suggests to combine the results of a functional in vitro MMR assay with those of an in silico analysis. Here, STEP2 is composed of the results of the in vitro MMR assay and two separate in silico assays derived from the SIFT and MAPP-MMR programs. Pathogenicity of a VUS was indicated by the in vitro MMR assay alone or together with the deleterious results obtained in silico as well as by deleterious results obtained through both of the in silico methods even in the absence of such indication by the in vitro MMR assay. Where pathogenicity in STEP2 was suggested by only one in silico analysis, STEP3 with further assays were required. Nonpathogenic VUS were distinguished by fully completed STEP2 assays with no indication of pathogenicity.

STEP3 of the assessment model [Couch et al., 2008] is composed of a set of laboratory experiments taken to further clarify the pathogenicity of the variations, where previous steps did not already do so. This panel of experiments was suggested to include studies of protein stability, protein interaction, and subcellular localization. STEP3 assays study specific fragments of the repair process complementing the in vitro MMR assay, which was performed in nuclear extracts and optimized to reveal even the slightest repair, and thus, not able to discover problems in subcellular localization or mild problems in protein stability or interactions. Here, the STEP3 assays differ slightly between the three genes but have been included for all variations for the verification of the three-step model. Together with one deficient result from an in silico assay, a decrease in variant protein expression, interaction, or localization was considered to be an indication of pathogenicity. Consequently, a single indication of pathogenicity in STEP3, although measuring different aspects of the protein function than the MMR assay, was deemed sufficient to confirm variant pathogenicity.

In order to verify the three-step model and determine the necessity and validity of all three steps in it, results and interpretations after STEP2 and STEP3 were compared. Finally, STEP1 data was considered together with these comparisons to further verify the indications of pathogenicity and to form a consensus for each step of the model.

## Results

### Verification of the Three-Step Model with *MLH1* Variations

Our results show that, if strong evidence from the family history, MSI and IHC results give an indication of LS and an *MLH1* variation has been detected in STEP1, STEP2 is often sufficient to confirm the pathogenicity of the variation. As seen in Table 1, when STEP2 results unanimously indicate pathogenicity (11/37), the expression and localization analyses of STEP3 serve to confirm the results and are hence descriptive but not essential for assessment. This is also apparent when MMR proficiency is unanimously indicated by all STEP2 assays as seen in 7/37 variations. Assuming that pathogenicity can be shown with the in vitro MMR assay alone (5/37) or with two deleterious in silico indications (4/37), STEP2 is sufficient to confirm the pathogenicity of 20/37 *MLH1* VUS. The former requisite is supported by VUS for which the in silico results are not obtainable, because all the five *MLH1* deletions (p.T45_I47delinsCF, p.E71del, p.I330del, p.P578_E632del, and p.E633_E663del) indicated to be pathogenic by the in vitro MMR assay, are confirmed to be so by STEP3, regardless of the lack of in silico results. Likewise, the 4 *MLH1* VUS (p.L550P, p.P648L, p.P648S, and p.654L) indicated to be pathogenic by both in silico analyses, but not with the MMR assay, are confirmed to be so by STEP3.

**Table 1:** Verification Data of *MLH1* Variations

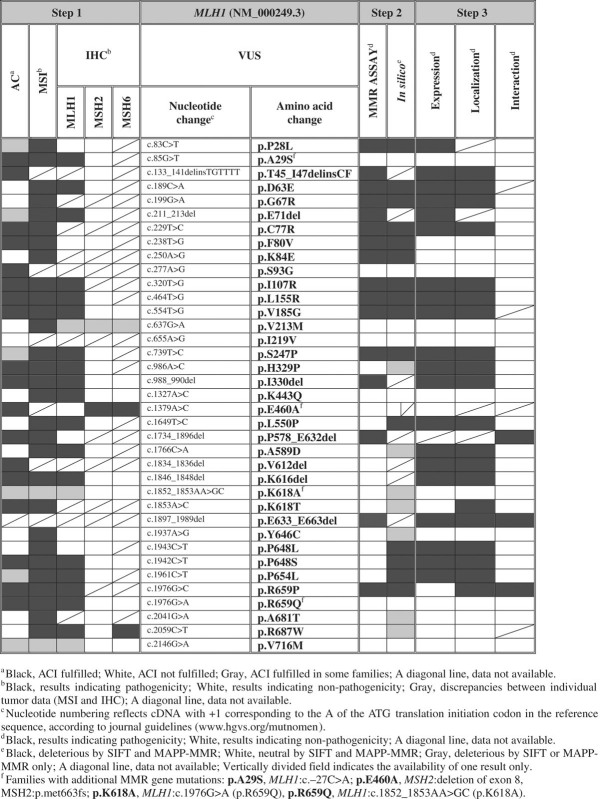

Nevertheless, in the absence of in silico results, or if pathogenicity is indicated by only one alignment analysis, the MMR proficiency is not sufficient to rule out pathogenicity. This is indicated by MMR proficient *MLH1* p.H329P, p.A589D, p.V612del, p.K616del, and p.K618T that display reduced expression and/or nuclear localization in STEP3. Generally, in such cases the expression assay suffices for STEP3. Especially, results from MLH1/PMS2 protein interaction analysis appear to have very little contribution toward the assessment of *MLH1* VUS. In contrast, four variants (p.K618A, p.Y646C, p.A681T, and p.R687W), in which the STEP2 differences are due to an indication of pathogenicity by only one in silico assay, the pathogenicity is not confirmed by STEP3. Similarly, for *MLH1*-p.E460A, deficiency indicated by only SIFT is not confirmed by STEP3.

The verification of the three-step model with the *MLH1* variations demonstrates that STEP3 often supports the deductions, which, however, can be made from STEP2 analyses in the case of 27/37 variations. Only in the case of MMR proficient variations with either discrepant or no data from both in silico methods, STEP3 is required for interpretation (10/37).

### Verification of the Three-Step Model with *MSH2* Variations

As with the variations affecting *MLH1*, when the MMR assay results agree with those obtained in silico*,* STEP3 is not necessary. This is seen in the case of 12 pathogenic and 7 nonpathogenic *MSH2* variations (Table 2). Pathogenicity indicated by the in vitro MMR assay result or with two deleterious in silico results confirms the pathogenicity of 15 VUS raising the total number of successfully assessed VUS in STEP2 to 22 out of 26. Notably, no indication of pathogenicity is seen in STEP3 only.

**Table 2:** Verification Data of *MSH2* Variations

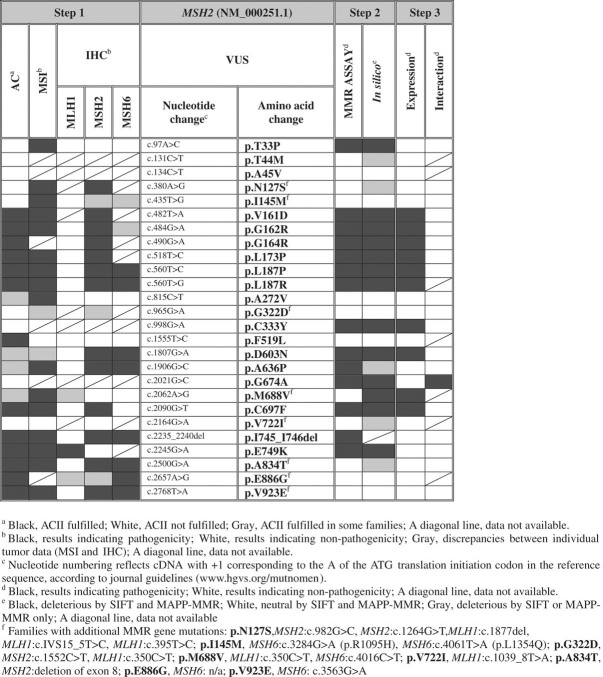

Unlike in the case of some *MLH1* variations, the pathogenicity of *MSH2* variations (p.T44M, p.N127S, p.V722I and p.A834T) indicated by only one in silico assay cannot be confirmed by STEP3 assays. *MSH2*-p.T44M has poor availability of clinical data, whereas the other three VUS (p.N127S, p.V722I, p.A834T) have been found in individuals with other MMR gene variations. Remarkably, a total of 8 of the 26 *MSH2* variations (p.N127S, p.I145M, p.G322D, p.M688V, p.V722I, p.A834T, p.E886G and p.V923E) have been found in carriers of other MMR gene variations. Furthermore, seven of these variations do not appear pathogenic, although three (*MSH2*-p.N127S, *MSH2*-p.V722I, and *MSH2*-p.A834T) are indicated to be deleterious by SIFT analysis. *MSH2*-p.M688V is assumed as pathogenic by both in silico results and decreased expression in STEP3 indicating protein instability even though MSH2 protein was detectable by IHC. Overall, the IHC data linked to these eight variations is either incomplete or in many cases in concordance with the additional variations found in the carriers.

The verification of the three-step model with the *MSH2* variations demonstrates that STEP2 is sufficient to assess the pathogenicity of 22/26 *MSH2* VUS. The confirming role of STEP3 is important in the four MMR proficient variations for which pathogenicity is suggested by only one in silico result. None of the pathogenicities indicated by only one in silico result is confirmed by STEP3.

### Verification of the Three-Step Model with MSH6 Variations

Even though none of the families carrying *MSH6* variations fulfilled the AC, the variations originate from suspected LS families with MSI-H tumor phenotypes. Only one of the 11 *MSH6* variations (p.E1193K) reliably indicates pathogenicity with the three-step approach of assessment (Table 3) as seen by the lack of MSH6 by IHC (STEP1), by in vitro MMR deficiency (STEP2, in silico results not available), and by reduced MSH2 interaction capability (STEP3). Reduced expression of *MSH6*-p.G566R (STEP3) suggests pathogenicity, which is supported by assays measuring its ability to stimulate ATPase activity [Cyr and Heinen, 2008; Kariola et al., 2002]. Nevertheless, MSH6-p.G566R does not appear pathogenic in STEP2.

**Table 3:** Verification Data of *MSH6* Variations

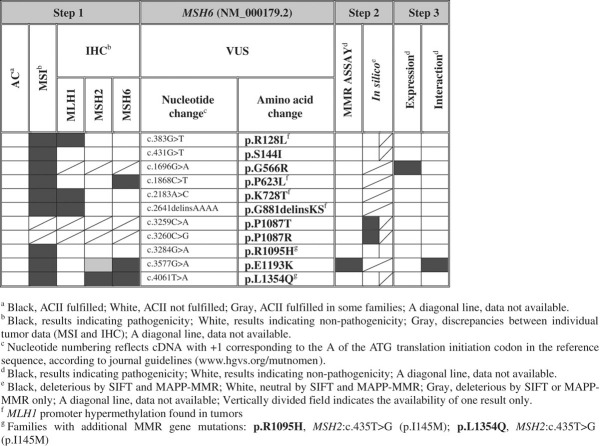

No indication of pathogenicity is detectable for three *MSH6* variations (p.R128L, p.K728T, p.G881delinsKS) notably, all of which lack MLH1 protein but not MSH6 (or MSH2) according to the IHC results. The MMR deficiencies of these tumors as well as that of *MSH6*-p.P623L have indeed been shown to be due to *MLH1* promoter hypermethylation [Kariola et al., 2004], suggesting that these four VUS are nonpathogenic. Moreover, as seen with 8 *MSH2* VUS, an additional MMR gene variation, a nonpathogenic *MSH2*-p.I145M variation has been found in both *MSH6*-p.R1095H and *MSH6*-p.L1354Q carriers. There appears to be no indication of *MSH6*-p.S144I pathogenicity; however, the in silico results were not obtainable. *MSH6*-p.P1087T and *MSH6*-p.P1087R on the other hand, are predicted to be deleterious by SIFT analysis although the MMR in vitro and STEP3 analyses do not detect defects in protein function.

Even though the 11 *MSH6* variations do not compose an ideal data set for the verification of the three-step model in assessing VUS pathogenicity, the importance of the interpretation of tumor IHC data prior to the identification of the VUS taken for further assessment is highlighted. Where the loss of MLH1 is detected by IHC, *MLH1* promotor hypermethylation analysis should be carried out prior to *MSH6* mutation analysis. The applicability of the model is further challenged by the lack of reliable in silico predictions for *MSH6* alterations and only if MSH6 deficiency is indicated by STEP1, or by the exclusion of other causative mutations, the verification of the three-step model is feasible.

### The Necessity of STEP3 in the Three-Step Model

**Figure 2:**
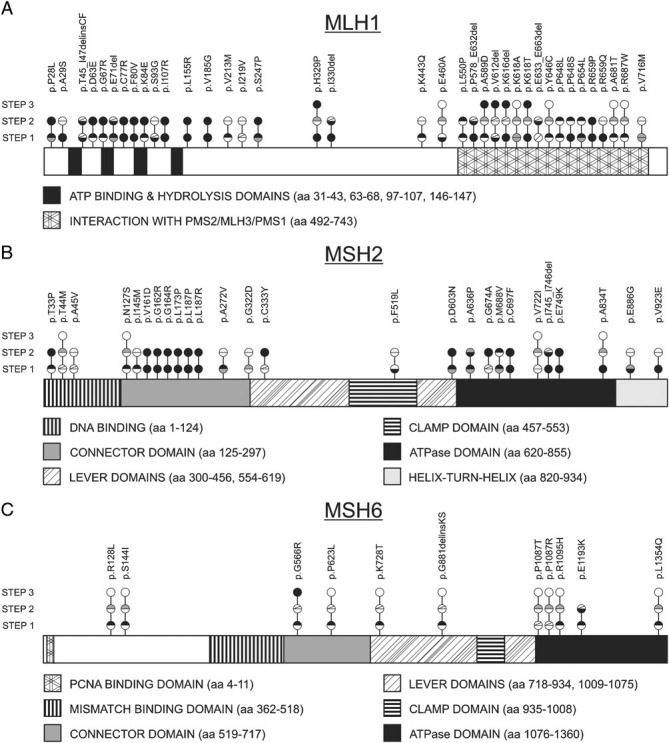
Schematic illustration of (**A**) MLH1 (**B**) MSH2, and (**C**) MSH6 showing the known functional domains, locations of the studied variations, and the amount of steps required for their assessment of pathogenicity. Each required step of the three-step assessment model is represented with a circle. STEP1 is divided to indicate the accordance of the family history with the Amsterdam criteria I/II (lower half: black, AC fulfilled; white, AC not fulfilled; gray, AC fulfilled by some families; diagonal line, data not available) and the MSI and IHC results of the tumor (upper half: black, MSI-H and/or reduced protein expression [IHC]; white, no MSI-H and no problems in protein expression (IHC); gray, contradicting data between several families; diagonal line, data not available). STEP2 is divided to indicate variant protein in vitro MMR activity (lower half: black, deficient; white, proficient) and in silico results (upper half: black, pathogenic effect predicted by SIFT and MAPP-MMR; white, neutral effect predicted by SIFT and MAPP-MMR; gray, discordant or only one result, either pathogenic or nonpathogenic available; diagonal line, data not available). STEP3 assay results are combined to assess VUS pathogenicity (black, results indicating pathogenicity; white, results indicating nonpathogenicity; gray, STEP inconclusive; diagonal line, data not available).

Only with 3/74 variations (*MLH1*-p.V612del, *MLH1*-p.K616del, and *MSH6*-p.G566R) STEP3 results provided information not already indicated by STEP2. Remarkably, all three variations lack in silico results. Hence, STEP3 is useful for the verification of the STEP2 assays, yet descriptive but often not necessary for the assessment of VUS pathogenicity. The verification of the model is indicated in terms of the amount of steps required for pathogenicity assessment of each VUS. Figure 2 demonstrates the collected results of each required assessment step for each gene and its variations. STEP3 of the assessment model across the variations of all three genes serves to confirm differences between the results of the previous assessments. Furthermore, STEP3 can be utilized to confirm or clarify causes of variant pathogenicity indicated by STEP2. STEP3 of the three-step model confirms the pathogenicity of 36 variations of which 31 is indicated by a reduction in expression. Where STEP3 is required, expression analysis should be the assay of choice, as only four variations indicate pathogenicity in STEP3 by localization or interaction analyses and not by the expression analysis.

## Discussion

Based on the application of 74 MMR gene variations and clinical data, the three-step assessment model seems to be a valuable tool for correctly identifying pathogenic MMR gene mutations, which in turn, permits predictive gene testing in the family and enables targeted cancer surveillance. The identification of the MMR gene for mutation analysis greatly relies on the comprehensive use of the patient's family history, MSI and IHC data. The absence of an MMR protein in IHC gives a good but not an absolute indication of the causative gene responsible for the MSI phenotype and subsequent tumorigenesis as indicated by the variations included in this study. Pathogenic *MSH2* mutations are shown to be highly associated with the lack of protein expression in IHC analyses [Mangold et al., 2005; Ollila et al., 2008], which is also frequently characterized by the absence of MSH6, the heterodimerization partner of MSH2 [Chang et al., 2000]. Furthermore, the sensitivity of IHC in predicting pathogenic *MSH6* mutations has been said to be as high as 90% [Hendriks et al., 2004]. However, IHC results demonstrating the lack of MLH1 expression may be misleading as MLH1 expression is often lost due to the hypermethylation of its promoter region [Kane et al., 1997]. In addition, the presence of a protein cannot be implied to indicate its functionality as pathogenicity can be caused by functional problems not affecting the stability of the protein [Mangold et al., 2005; Raevaara et al., 2005].

The application of the 74 variants to the three-step assessment model suggests that pathogenicity is reliably indicated by the STEP2 in vitro MMR assay as supported by other functional assays in STEP3. Nonetheless, when no indication of pathogenicity is seen in the in vitro MMR assay the importance of computational methods become apparent. In silico methods have been shown to have a high predictive value (88.1%) when four different methods are in agreement [Chan et al., 2007] and alignments are manually revised [Tavtigian et al., 2008a]. As the model proposed by Couch et al. [2008] considers the in vitro MMR and in silico assay results in a single step, we combined results from two in silico approaches previously shown to be appropriate for MMR gene variations [Chao et al., 2008; Ollila et al., 2006; Tavtigian et al., 2008b], with those obtained from the in vitro MMR assay to verify the model and the necessity of STEP3. A total of 28/63 *MLH1* and *MSH2* variations are indicated as pathogenic by both in silico methods and in 24 of them pathogenicity is further supported by STEP3 results. Our results also suggest that in most cases where discrepancies between SIFT and MAPP-MMR results are seen, the in vitro MMR proficiency should be assumed correct. A single in silico result suggesting deficiency is often linked to other ambiguities associated to the variation and is not sufficient to characterize the MMR variation alone as seen in the case of seven *MLH1*, four *MSH2,* and two *MSH6* variations. Of these, only three *MLH1* variations were confirmed to be pathogenic by STEP3 assays. Overall, STEP3 of the assessment model is not required in cases where it does not provide information not already revealed by STEP2. Variant deficiency can be indicated by the in vitro MMR assay alone or by both in silico analyses; hence, STEP2 suffices for the characterization of most *MLH1* (27/37) and *MSH2* (22/26) variations. The 11 *MSH6* variations applied to verify the model present with atypical family background and should hence be assessed with scrutiny. Regardless of the limited selection of *MSH6* variations the applicability of the three-step model to the assessment of *MSH6* variations is not ruled out—merely more attention to STEP1 is called for in order to eliminate phenotypes caused by other MMR genes. As our results suggest, the MSI-H phenotype in 3/11 tumors from *MSH6* VUS carriers is more likely to be linked to the *MLH1* promoter hypermethylation than *MSH6* variations found in the families, and thus, if *MLH1* expression is lost in the tumor, its promoter hypermethylation should be assayed before the more challenging and time consuming functional assays.

Only two *MLH1* (p.V612del and p.K616del) and one *MSH6* (p.G566R) variations of the total 74 (4%) indicate pathogenicity in STEP3 for the first time. Of these, p.K616del pathogenicity is supported by the AC fulfillment, MSI and IHC data, whereas p.V612del pathogenicity is supported by only the AC fulfillment. Here, the lack of indication of pathogenicity already in STEP2 is probably due to the lack of in silico data, which is unfortunately the case for in frame deletions. We also want to acknowledge that the in vitro MMR assay was carried out using parameters, which maintain the amount of variant protein at optimal levels for detecting even the slightest repair. In the future, the assay could also be titrated to allow the detection of less prominent functional defects, possibly facilitating the assessment of carboxyl terminal *MLH1* variations, which nevertheless are currently recognized by the combination of two in silico analyses in STEP2.

When a variation does not appear to be causative of the LS phenotype, the presence of other predisposing variations should be considered. Carriers of 14 VUS (four *MLH1*, eight *MSH2,* and two *MSH6*) included in this verification were reported to also carry other MMR gene variations. Unsurprisingly, only 1 (*MSH2*-p.M688V) of the 14 VUS could be considered as pathogenic, although the other mutations, *MLH1* (p.T117M) and *MSH6* (p.A1889V), identified in the *MSH2*-p.M688V carriers may also contribute toward the LS phenotype [Christensen et al., 2008, 2009]. Another problematic VUS in terms of assessment of pathogenicity is *MLH1*-p.K618A, because seven families carrying the variation show extremely variable phenotypes in terms of MSI and IHC. Even though its slightly decreased ability to interact with PMS2 has been reported [Guerrette et al., 1999; Kondo et al., 2003], p.K618A does not appear to be pathogenic by STEP2 and STEP3 assays. Furthermore, one of the *MLH1*-p.K618A families was also reported to carry *MLH1*-p.R659Q missense mutation affecting a codon highly linked to the aberrant splicing and subsequent skipping of exon 17 (p.E633_E663del) [Kohonen-Corish et al., 1996; Nyström-Lahti et al., 1999]. Interestingly *MLH1*-p.R659Q itself does not appear to be pathogenic, suchs as other VUS (p.R659P) in the same codon. Skipping of exon 17 (*MLH1*-p.E633_E663del) is, however, shown to cause MMR deficiency [Nyström-Lahti et al., 2002].

According to our verification, the three-step assessment model is a logical and useful tool for the assessment of the pathogenicity of MMR variations as demonstrated with the 74 VUS and their clinical, laboratory, and computational data. When both the in vitro MMR and the in silico assay results are available, STEP2 of the assessment model seems to be sufficient to assess the pathogenicity of most *MLH1* and *MSH2* variations. Furthermore, STEP2 also suffices to indicate nonpathogenicity to the same extent as STEP3, and is hence also important in guiding the reassessment of the cause of LS susceptibility in a family. The ultimate aim should be to obtain a classification of the VUS based on the probability of being pathogenic, as proposed by Plon et al. [2008] using five probability classes ranging from definitely pathogenic to not pathogenic or of no clinical significance. Although the results of this work are not yet sufficient to allow application of this specific approach in the clinical setting, and at this stage, without determined cutoffs for separate assays, the model does not allow to consider all five classes, the results are promising, and thus encourage the use of the model for a comprehensive validation study against a set of VUS that have been defined as clearly pathogenic or neutral.
